# Supplementary material for: Fe3O4@COF(TAPT–DHTA) Nanocomposites as Magnetic Solid-Phase Extraction Adsorbents for Simultaneous Determination of 9 Mycotoxins in Fruits by UHPLC–MS/MS
Source: Toxins (Basel). 2023 Feb 1;15(2):117. doi: 10.3390/toxins15020117 (PMC9966527; doi:10.3390/toxins15020117)
Supplement: Supplementary file 1 [file toxins-15-00117-s001.zip › toxins-2170282-supplementary.pdf]

Supplementary Materials: Fe_3O_4@COF(TAPT−DHTA) nanocomposites as magnetic solid-phase extraction adsorbents for simultaneous determination of 9 mycotoxins in fruits by UHPLC−MS/MS

Jie Wang [1,2,†], Qingwen Huang [2,†], Wenbo Guo [2], Dakai Guo [2], Zheng Han [2] and Dongxia Nie [1,2,*]

[1] School of Health Science and Engineering, University of Shanghai for Science and Technology, Shanghai 200093, China
[2] Institute for Agro-Food Standards and Testing Technology, Shanghai Academy of Agricultural Sciences, Shanghai 201403, China
* Correspondence: niedongxia@saas.sh.cn; Tel.: +86-21-37196975
† These authors contributed equally to the study.

Table S1. Structure and physicochemical parameters of 9 mycotoxins

Table S2. Mass spectrometry parameters of 9 mycotoxins

Table S3. Significance analysis of adsorbed solution

Table S4. Significance analysis of pH value of adsorbed solution

Table S5. Significance analysis of NaCl concentration in adsorbed solution

Table S6. Significance analysis of Adsorption time

Table S7. Significance analysis of adsorbent amount

Table S8. Significance analysis of elution kinds

Table S9. Significance analysis of Elution Volume

Table S10. Significance analysis of Elution time

Table S11. Comparison of the Fe_3O_4@COF(TAPT−DHTA) based MSPE method developed in this study with the sample pretreatment approaches reported in the previous studies.

Table S12. Contamination levels of 9 mycotoxins in five fruits (tomato, strawberry, watermelon, melon, hawthorn)

Table S13. Comparison between the developed UHPLC−MS/MS method and the reference methods by using tomato sample No. 7,14 and 20 ($\mu g\ kg^{-1}$)

Figure S1. UV-Vis spectra of targeted mycotoxins, Fe_3O_4@COF(TAPT−DHTA) and mycotoxin/Fe_3O_4@COF(TAPT−DHTA)

Figure S2. Comparison of the purification efficiency of 9 mycotoxins in the spiked tomato sample by 8 candidate elution solutions

Figure S3. Effects of eluent volume on Fe_3O_4@COF (TAPT−DHTA) MSPE process

Figure S4. MRM chromatography of 9 mycotoxins in (A)solvent standard solution and (B) matrix standard solution (100 μg kg^{-1}).

Table S1. Structure and physicochemical parameters of 9 mycotoxins

Mycotoxins	Structure	Molecular weight	pKa	Molecular Formula	Hydrogen Bond Donor Count	Hydrogen Bond Acceptor Count
Tenuazonic acid (TeA)	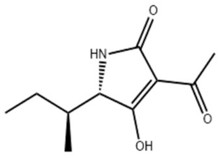	197.23	4.50±1.00	$C_{10}H_{15}NO_3$	2	3
Alternariol (AOH)	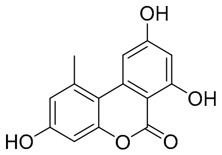	258.23	7.16±0.20	$C_{14}H_{10}O_5$	3	5
Tentoxin (TEN)	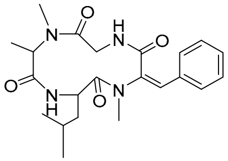	414.50	_	$C_{22}H_{30}N_4O_4$	2	4
Alternariol monomethyl ether (AME)	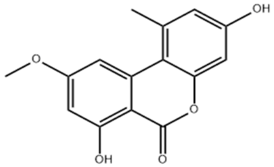	272.25	7.00±0.20	$C_{15}H_{12}O_5$	2	5
Altenuene (ALT)	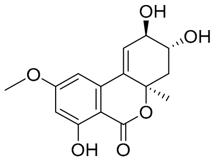	292.28	7.41±0.70	$C_{15}H_{16}O_6$	3	6

Mycotoxins	Structure	Molecular weight	pKa	Molecular Formula	Hydrogen Bond Donor Count	Hydrogen Bond Acceptor Count
Altenusin (ALS)	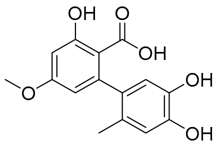	290.27	3.08±0.34	$C_{15}H_{14}O_6$	4	6
Aflatoxin B_1 (AFB_1)	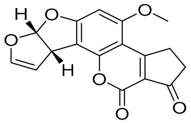	312.27	_	$C_{17}H_{12}O_6$	0	6
Ochratoxin A (OTA)	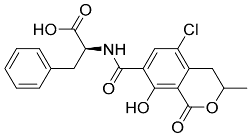	403.81	3.40±0.10	$C_{20}H_{18}ClNO_6$	3	6
Zearalenone (ZEN)	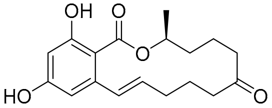	318.40	7.58±0.40	$C_{18}H_{22}O_5$	2	5

Table S2. Mass spectrometry parameters of 9 mycotoxins

Mycotoxins	Precursor Ions (m/z)	Retention time (min)	Product Ions (m/z)	Dwell Time (s)	Cone Voltage (V)	Collision Energy (eV)
TeA	198.1 $[M+H]^+$	1.75	153.1058 [a]	0.005	42	16
			125.0214 [b]	0.005	42	12
AOH	259.0 $[M+H]^+$	4.45	185.1184 [a]	0.005	64	28
			213.1284 [b]	0.005	64	24
AME	273.0 $[M+H]^+$	5.65	258.0000 [a]	0.012	54	26
			128.0600 [b]	0.012	54	25
ALT	292.9 $[M+H]^+$	3.64	257.0693 [a]	0.005	30	14
			275.0738 [b]	0.005	30	8
TEN	415.5 $[M+H]^+$	4.76	132.1335 [a]	0.005	14	34
			256.2291 [b]	0.005	14	34
ALS	288.8 $[M-H]^-$	2.62	245.0728 [a]	0.005	8	20
			230.0566 [b]	0.005	45	17
AFB_1	313.2 $[M+H]^+$	3.78	241.1575 [a]	0.009	74	36
			284.9710 [b]	0.009	74	22
OTA	404.3 $[M+H]^+$	4.28	239.0344 [a]	0.005	30	34
			221.0280 [b]	0.005	30	22

ZEN	317.2 [M-H]$^-$	5.33	175.0464 [a]	0.012	56	26
			131.0360 [b]	0.012	56	24

[a] quantitation ion; [b] qualifier ions

Table S3. Significance analysis of adsorbed solution

Mycotoxins	Adsorption Solution (Recovery±SD %)					
	0%	1%	2%	3%	4%	5%
TeA	67.88±4.55^{b}	82.77±5.31^{a}	61.51±6.51^{bc}	56.64±3.63^{cd}	58.52±6.02^{c}	48.81±2.21^{d}
AOH	74.06±3.15^{b}	104.18±3.60^{a}	69.66±1.62^{bc}	74.10±5.59^{b}	67.32±6.41^{bc}	61.48±4.60^{c}
AME	81.23±10.28^{b}	99.62±5.91^{a}	80.73±11.58^{b}	79.31±4.54^{b}	54.19±6.29^{c}	40.48±5.12^{c}
TEN	69.31±5.76^{b}	87.71±12.93^{a}	42.84±2.48^{c}	34.68±3.93^{cd}	28.26±4.66^{de}	20.79±2.74^{e}
ALT	65.74±3.22^{b}	87.50±8.3^{a}	34.76±1.79^{c}	28.58±4.6^{cd}	25.53±1.18^{d}	20.71±1.57^{d}
ALS	63.29±4.93^{b}	85.81±12.76^{a}	53.21±6.89^{b}	33.83±3.17^{c}	20.70±2.87^{d}	15.47±2.86^{d}
AFB_1	74.50±7.50^{ab}	79.15±9.81^{ab}	81.32±8.69^{a}	72.99±9.95^{ab}	64.18±9.76^{bc}	55.54±5.03^{c}
OTA	87.73±3.66^{a}	90.71±2.66^{a}	79.62±4.22^{ab}	79.86±14.06^{ab}	60.09±11.08^{bc}	46.78±8.03^{c}
ZEN	77.34±2.40^{bc}	95.94±6.18^{a}	91.45±5.87^{ab}	85.69±10.75^{ab}	65.70±12.43^{cd}	54.05±4.43^{d}

Table S4. Significance analysis of pH value of adsorbed solution

Mycotoxins	pH of Adsorption Solution (Recovery±SD %)					
	pH=3	pH=4	pH=5	pH=6	pH=7	pH=8
TeA	62.15±10.15^{b}	83.01±4.11^{a}	61.26±7.34^{b}	54.28±2.44^{bc}	48.93±3.32^{c}	50.50±2.89^{c}
AOH	83.49±7.06^{ab}	95.99±7.95^{a}	94.92±1.23^{a}	83.86±4.26^{ab}	79.01±8.35^{ab}	71.14±1.11^{b}
AME	94.96±7.36^{a}	104.51±5.25^{a}	83.36±4.24^{ab}	66.26±0.87^{bc}	51.31±11.75^{c}	47.87±11.26^{c}
TEN	74.34±5.19^{b}	89.95±6.48^{a}	92.88±4.96^{a}	81.03±2.08^{ab}	54.14±1.31^{c}	50.55±4.46^{c}
ALT	57.45±3.82^{b}	87.64±9.59^{a}	68.03±1.13^{b}	68.52±8.17^{b}	67.94±3.59^{b}	62.89±11.75^{b}
ALS	64.06±9.09^{b}	82.81±4.02^{a}	83.79±1.88^{a}	63.01±5.34^{b}	54.50±0.88^{c}	51.20±1.70^{c}
AFB_1	57.98±1.04^{b}	76.42±3.92^{a}	55.95±0.70^{b}	39.09±0.62^{c}	39.30±3.25^{c}	36.93±4.58^{c}
OTA	50.43±3.84^{b}	88.29±0.93^{a}	53.94±0.62^{b}	39.42±1.07^{c}	41.15±5.64^{c}	28.23±1.07^{d}
ZEN	63.94±3.70^{b}	92.12±6.32^{a}	62.07±5.13^{b}	40.19±2.91^{c}	34.67±3.86^{c}	33.96±0.86^{c}

Table S5. Significance analysis of NaCl concentration in adsorbed solution

Mycotoxins	Concentration of NaCl (Recovery±SD %)					
	0 mg mL^{-1}	2 mg mL^{-1}	4 mg mL^{-1}	6 mg mL^{-1}	8 mg mL^{-1}	10 mg mL^{-1}
TeA	85.10±10.52^{a}	76.62±1.64^{b}	67.87±3.04^{c}	62.54±1.39^{c}	59.34±1.33^{c}	49.91±2.48^{d}
AOH	103.11±5.38^{a}	95.00±7.56^{a}	81.63±3.99^{ab}	71.01±2.99^{b}	59.63±3.05^{bc}	43.69±4.25^{c}
AME	100.28±5.87^{a}	95.88±2.09^{a}	61.61±4.72^{b}	54.77±10.44^{bc}	47.84±1.35^{c}	43.84±7.11^{c}
TEN	94.67±6.53^{a}	73.01±5.57^{b}	69.21±1.79^{b}	58.18±4.38^{c}	55.36±3.71^{cd}	47.92±3.30^{d}
ALT	83.22±2.68^{a}	69.68±5.56^{b}	63.43±2.09^{c}	68.46±1.48^{bc}	72.07±4.47^{b}	54.96±0.57^{d}
ALS	84.88±8.35^{a}	75.66±4.23^{a}	60.68±5.88^{b}	55.42±4.90^{b}	54.07±2.97^{b}	59.46±4.12^{b}
AFB_1	76.83±3.76^{a}	65.97±7.32^{b}	64.89±3.53^{b}	58.69±6.10^{bc}	49.10±1.57^{cd}	43.52±5.01^{d}
OTA	87.43±2.43^{a}	61.69±0.50^{b}	59.38±3.17^{b}	48.51±6.29^{c}	35.55±5.50^{d}	36.71±2.77^{d}
ZEN	94.10±6.01^{a}	62.32±7.32^{b}	49.90±5.80^{c}	41.82±0.72^{cd}	35.48±2.34^{de}	29.85±1.15^{e}

Table S6. Significance analysis of Adsorption time

Mycotoxins	Adsorption time (Recovery±SD %)				
	2 min	4 min	6 min	8 min	10 min
TeA	22.73±5.42^{d}	49.39±5.19^{c}	72.92±8.54^{b}	88.30±11.50^{a}	88.79±4.91^{a}
AOH	41.34±5.65^{d}	59.93±4.12^{c}	82.08±7.92^{b}	96.43±4.57^{a}	95.80±7.52^{a}
AME	32.58±2.22^{d}	46.02±2.27^{c}	74.66±1.91^{b}	96.58±1.55^{a}	99.66±4.02^{a}
TEN	31.00±1.00^{d}	62.92±1.10^{c}	74.50±8.64^{b}	95.97±0.72^{a}	92.66±8.90^{a}
ALT	53.48±4.49^{b}	76.77±9.62^{a}	88.26±8.01^{a}	85.46±7.79^{a}	93.18±1.74^{a}
ALS	49.84±3.18^{b}	61.23±8.91^{b}	79.39±4.90^{a}	89.42±1.79^{a}	93.76±9.50^{a}
AFB_1	19.75±11.71^{b}	31.63±6.87^{b}	64.74±0.31^{a}	78.16±2.73^{a}	77.41±4.22^{a}
OTA	49.23±8.70^{c}	65.93±5.84^{b}	72.23±7.82^{b}	91.29±6.68^{a}	86.36±8.04^{a}
ZEN	29.34±1.29^{c}	36.57±1.91^{c}	58.31±5.81^{b}	94.87±7.99^{a}	101.09±5.72^{a}

Table S7. Significance analysis of adsorbent amount

Mycotoxins	The amount of adsorbent (Recovery±SD %)				
	10 mg	15 mg	20 mg	25 mg	30 mg
TeA	71.26±4.03^{c}	85.57±4.75^{ab}	90.58±8.38^{a}	88.27±1.05^{ab}	75.02±2.30^{bc}
AOH	59.90±2.87^{d}	69.81±3.21^{c}	96.95±2.56^{a}	72.97±1.18^{bc}	74.39±1.13^{b}
AME	63.38±2.82^{c}	75.14±0.26^{b}	96.49±5.08^{a}	83.07±7.95^{b}	76.78±8.78^{b}
TEN	68.41±3.44^{b}	70.17±1.49^{b}	87.73±6.07^{a}	69.85±0.94^{b}	67.92±0.61^{b}
ALT	68.79±3.66^{c}	75.97±9.37^{bc}	90.63±3.29^{a}	83.79±2.16^{ab}	81.46±2.58^{ab}
ALS	64.40±1.99^{b}	72.28±5.09^{b}	82.32±8.99^{a}	76.08±4.34^{b}	79.34±5.31^{b}
AFB_1	35.83±0.99^{c}	54.81±0.68^{b}	75.26±0.81^{a}	73.63±1.28^{a}	77.81±6.10^{a}
OTA	45.47±4.09^{b}	48.52±1.38^{b}	83.80±7.59^{a}	72.22±5.67^{a}	76.53±7.57^{a}
ZEN	58.40±6.36^{c}	75.73±0.92^{b}	97.18±11.02^{a}	86.47±3.09^{ab}	84.37±10.31^{ab}

Table S8. Significance analysis of elution kinds

Mycotoxins	The kinds of Elution solvent (Recovery±SD %)							
	A	B	C	D	E	F	G	H
TeA	81.93±3.98^{b}	45.03±7.73^{d}	79.90±0.52^{b}	55.31±5.38^{cd}	68.78±1.74^{bc}	72.75±6.43^{b}	106.28±3.42^{a}	66.81±8.84^{bc}
AOH	99.82±9.16^{ab}	103.53±6.22^{ab}	94.33±1.87^{abc}	93.29±6.94^{bc}	81.73±8.30^{cd}	82.12±1.25^{cd}	107.33±5.08^{a}	76.05±2.60^{d}
AME	98.92±6.83^{a}	72.39±2.97^{cde}	70.19±2.47^{de}	75.16±1.97^{cd}	85.18±0.73^{b}	78.83±6.26^{bc}	93.64±6.23^{a}	65.49±2.96^{e}
TEN	91.04±7.38^{a}	66.18±4.34^{d}	74.69±3.87^{cd}	89.22±2.46^{ab}	80.64±0.77^{bc}	78.20±5.03^{c}	83.33±7.55^{abc}	77.78±4.42^{c}
ALT	89.25±2.61^{a}	52.42±8.83^{bcd}	65.96±5.28^{bc}	60.23±5.22^{bc}	64.08±4.38^{b}	47.28±7.59^{cd}	90.35±5.02^{a}	44.5±6.07^{d}
ALS	86.21±3.99^{a}	67.46±3.54^{bc}	76.44±0.72^{ab}	76.22±4.82^{ab}	72.05±1.002^{abc}	38.36±6.47^{d}	86.62±2.75^{a}	58.69±5.44^{c}
AFB_1	80.86±1.53^{a}	70.52±8.59^{b}	64.74±0.31^{bc}	61.49±2.73^{c}	40.19±2.51^{de}	35.23±0.64^{e}	72.19±8.54^{b}	45.04±0.89^{d}
OTA	87.69±5.48^{a}	71.48±6.72^{bc}	61.12±1.89^{c}	80.18±2.87^{ab}	62.47±2.15^{bc}	43.32±1.42^{d}	87.36±1.42^{a}	44.39±1.85^{d}
ZEN	94.24±3.43^{a}	69.91±1.91^{b}	65.53±0.51^{b}	72.65±4.95^{b}	67.76±5.72^{b}	66.07±0.73^{b}	68.23±8.63^{b}	53.85±7.39^{c}

(A) Methanol, (B) Acetonitrile, (C) Methanol/Formic acid (99/1), (D) Acetonitrile/Formic acid (99/1), (E) Methanol/Acetonitrile/Formic acid (50/49/1), (F) Methanol/Acetonitrile/Ammonium hydroxide (50/49/1), (G) Methanol/Acetonitrile/Formic acid (80/19/1), (H) Methanol/Acetonitrile/Ammonium hydroxide (80/19/1)

Table S9. Significance analysis of Elution Volume

	3 mL	9 mL	15 mL
TeA	88.15±7.30[a]	80.53±3.95[a]	80.65±7.49[a]
AOH	99.69±2.73[a]	84.97±2.63[a]	93.91±1.66[a]
AME	96.80±3.02[a]	104.35±5.29[a]	98.41±3.92[a]
TEN	88.99±7.09[ab]	82.04±3.49[b]	94.90±2.21[a]
ALT	83.41±3.99[a]	71.26±3.24[a]	67.62±7.81[a]
ALS	81.39±8.74[a]	86.55±4.75[a]	83.93±2.73[a]
AFB_1	78.40±5.08[a]	75.67±0.34[a]	76.39±1.20[a]
OTA	88.85±3.48[a]	89.31±7.16[a]	81.88±6.15[a]
ZEN	98.6±5.71[ab]	103.88±10.15[a]	84.70±3.06[b]

Table S10. Significance analysis of Elution time

Mycotoxins	Elution time (Recovery±SD %)				
	1 min	2 min	3 min	4 min	5 min
TeA	38.49±3.30[c]	65.98±2.38[b]	70.14±3.29[b]	91.19±1.98[a]	90.06±2.70[a]
AOH	42.29±6.55[c]	76.57±7.40[b]	103.03±10.37[a]	101.99±6.40[a]	99.96±5.85[a]
AME	48.12±2.97[c]	64.14±5.67[b]	99.39±7.20[a]	103.62±2.06[a]	100.29±5.14[a]
TEN	37.85±0.93[c]	63.64±0.27[b]	85.58±0.87[a]	86.26±0.60[a]	83.34±1.44[a]
ALT	41.53±4.79[c]	54.18±5.08[b]	82.20±2.27[a]	88.02±7.98[a]	83.44±6.11[a]
ALS	40.69±2.85[b]	65.91±6.75[a]	72.07±4.50[a]	80.22±9.80[a]	81.76±5.85[a]
AFB_1	33.26±4.93[c]	52.23±8.38[c]	65.50±6.30[a]	75.08±3.05[a]	77.34±4.96[a]
OTA	32.77±1.78[c]	59.66±6.41[b]	64.28±7.4$_b$	90.96±1.50[a]	87.81±3.07[a]
ZEN	41.35±1.54[c]	51.78±3.74[bc]	59.41±2.97[b]	96.71±8.00[a]	97.80±3.68[a]

Table S11. Comparison of the Fe_3O_4@COF(TAPT−DHTA) based MSPE method developed in this study with the sample pretreatment approaches reported in the previous studies.

Mycotoxins	Matrix	Sorbent	Sample pretreatment	Analytical technique	Adsorption/ Elution time of MSPE (min)	Recovery (%)	LOD (μg kg^{-1})	Ref.
FB_1, ZON, OTA	maize oil, rapeseed oil, soybean oil	Fe_3O_4@nSiO_2@mSiO_2	MSPE	UPLC−MS/MS	10/5	89.40-97.10	0.08-1.03	[1]
AFB_1, AFB_2, AFG_1, AFG_2, OTA, OTB	vegetable oils	PDA@Fe_3O_4-MWCNTs	MSPE	HPLC−FLD	10/3	70.15-89.25	0.20-0.50	[2]
AFB_1, ZON	Wheat flour	Fe_3O_4-MWCNTs-NH_2	MSPE	HPLC	25/15	88.80-96.00	0.15-0.24	[3]
AFB_1, AFG_1, STER, ZEN, OTA	licorice	Fe_3O_4@PDA/MIL-101(Cr)	MSPE	UHPLC−MS/MS	30/2	78.53-116.28	0.01–0.09	[4]
AOH, AME, TEN, TeA	jujube	$MgSO_4$, NaCl	QuEChERS	UPLC−MS/MS		83.50-109.60	0.14-0.26	[5]
AOH, AME, TEN, TeA	tomato, fruit-based products	$MgSO_4$, NaCl	QuEChERS	LC−MS/MS		98.80-108.90	0.25-20.00	[6]
AOH, AME, TEN, TeA, ALT	wolfberry	C18	QuEChERS	UPLC−MS/MS		73.80-111.50	0.07-0.24	[7]
9 mycotoxins	Tomato, Strawberry, Watermelon, Melon, Hawthorn	Fe_3O_4@COF(TAPT−DHTA)	MSPE	UHPLC−MS/MS	8/4	74.25-111.75	0.01-0.50	This work

Table S12. Contamination levels of 9 mycotoxins in five fruits (tomato, strawberry, watermelon, melon, hawthorn)

Mycotoxins	Tomato		Strawberry		Watermelon		Melon		Hawthorn	
	Positive/Total samples	Content range (μg kg⁻¹)	Positive/Total samples	Content range（μg kg⁻¹）	Positive/Total samples	Content range（μg kg⁻¹）	Positive/Total samples	Content range（μg kg⁻¹）	Positive/Total samples	Content range（μg kg⁻¹）
TeA	10/20	3.8-6.5	9/20	1.9-5.6	0/20	ND [a]	1/20	＜LOQ	0/20	ND
AOH	2/20	3.05-4.0	3/20	4.9-20.0	1/20	＜LOQ	0/20	ND	5/20	3.7-14.2
AME	0/20	ND	0/20	ND	0/20	ND	1/20	1.4	6/20	10.9-16.8
TEN	1/20	18.2	0/20	ND	0/20	ND	0/20	ND	3/20	0.6-1.3
ALT	8/20	2.2-44.5	11/20	3.4-54.8	5/20	29.5-56.3	10/20	43.4-123.7	9/20	38.8-190.4
ALS	1/20	1.88	3/20	＜LOQ	0/20	ND	4/20	1.3-5.9	2/20	4.3-8.1
AFB_1	0/20	ND	0/20	ND	0/20	ND	0/20	ND	0/20	ND
OTA	0/20	ND	0/20	ND	0/20	ND	0/20	ND	0/20	ND
ZEN	0/20	ND	0/20	ND	0/20	ND	0/20	ND	0/20	ND

[a]: means not detected

Table S13. Comparison between the developed UHPLC−MS/MS method and the reference methods by using tomato sample No. 7,14 and 20 (μg kg^{-1})

	Samples	TeA	AOH	AME	ALT	AFB_1	OTA	TEN	ALS	ZEN
Current method	7		3.05±0.08[a]		9.09±0.19[a]					
	14				7.65±0.27[a]			18.20±0.26[a]		
	20				13.79±0.75[a]				1.88±0.12[a]	
Reference method*	7		2.89±0.18[a]		9.28±0.97[a]					
	14				8.39±0.53[a]			17.09±1.14[a]		
	20				13.62±3.06[a]				2.01±0.53[a]	

*Reference method: SN/T 4259-2015

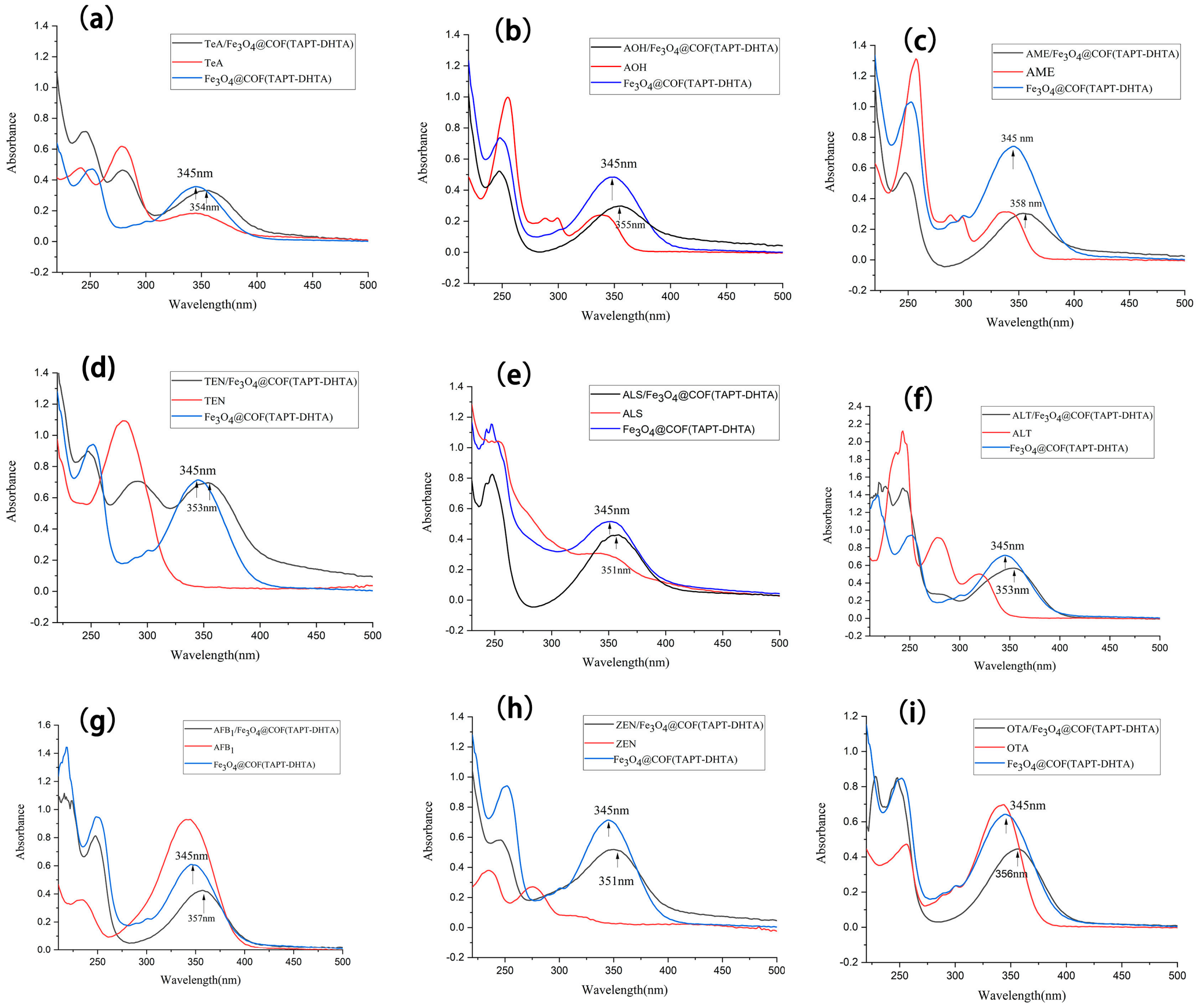

Figure S1. UV-Vis spectra of targeted mycotoxins, Fe_3O_4@COF(TAPT−DHTA) and mycotoxin/Fe_3O_4@COF(TAPT−DHTA)

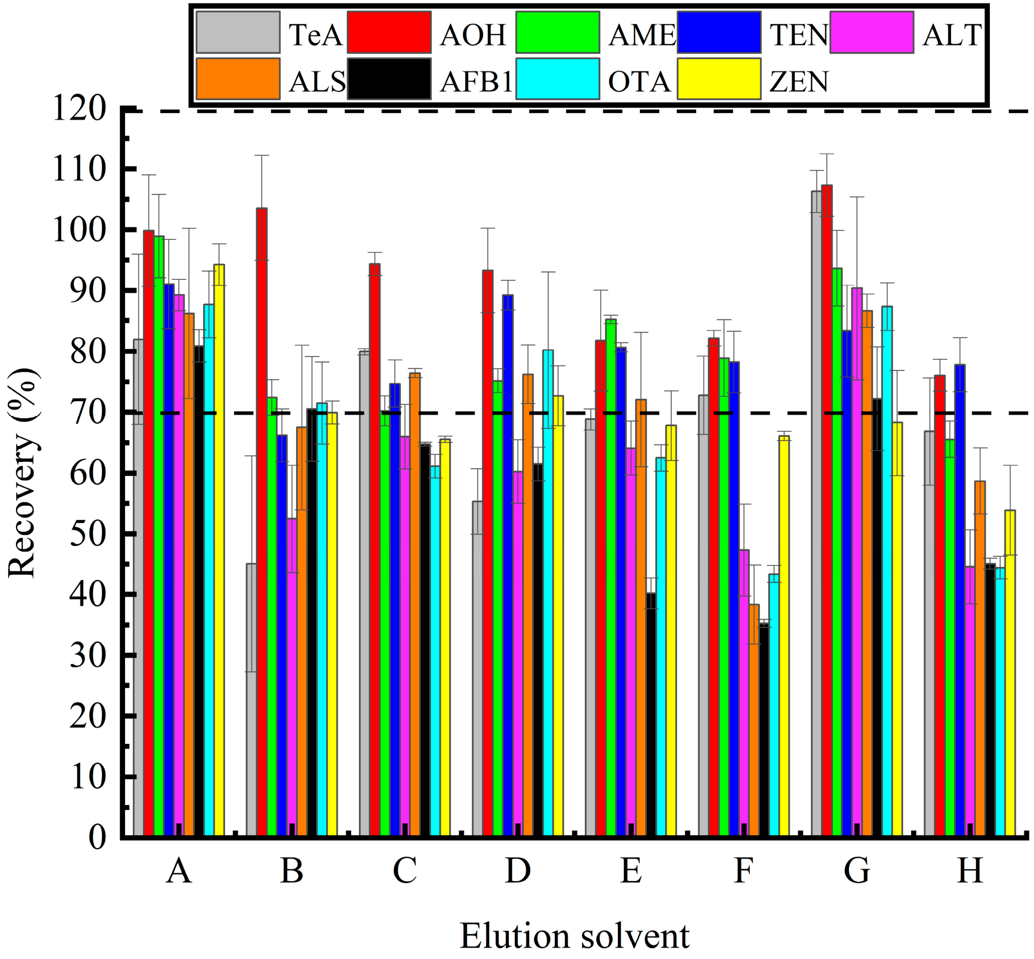

Figure S2. Comparison of the purification efficiency of 9 mycotoxins in the spiked tomato sample by 8 candidate elution solutions，(A) Methanol, (B) Acetonitrile, (C) Methanol/Formic acid (99/1), (D) Acetonitrile/Formic acid (99/1), (E) Methanol/Acetonitrile/Formic acid (50/49/1), (F) Methanol/Acetonitrile/Ammonium hydroxide (50/49/1), (G) Methanol/Acetonitrile/Formic acid (80/19/1), (H) Methanol/Acetonitrile/Ammonium hydroxide (80/19/1)

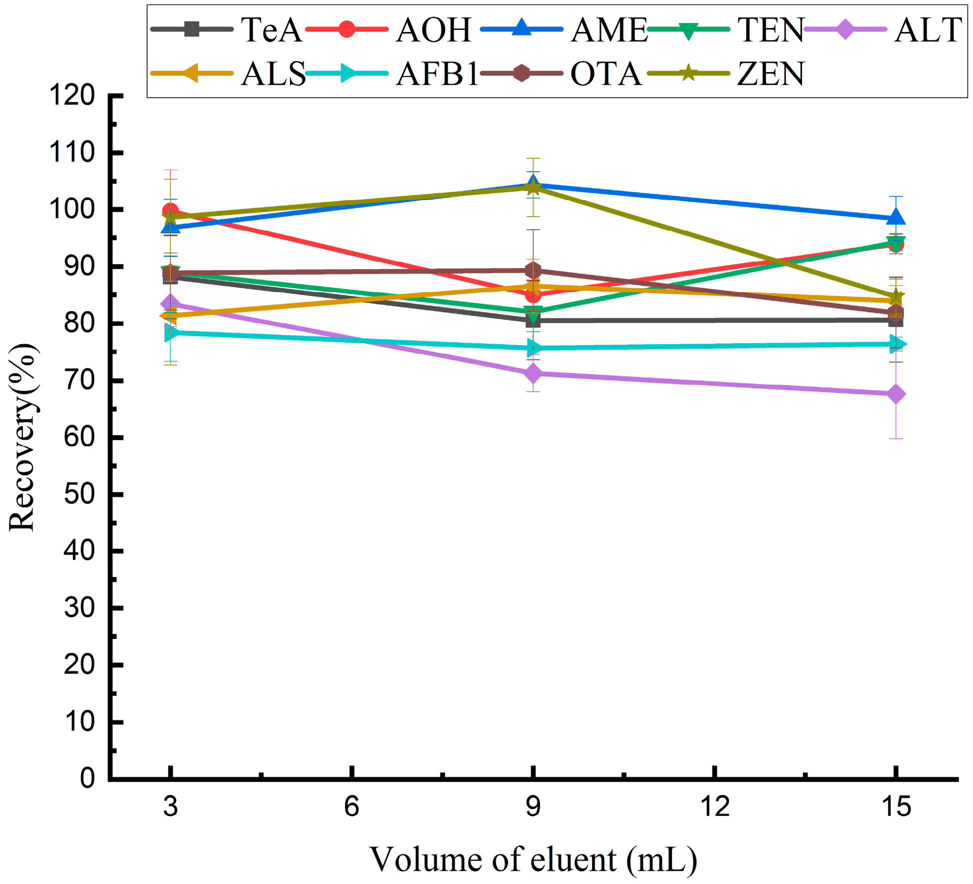

Figure S3. Effects of eluent volume on Fe_3O_4@COF (TAPT−DHTA) MSPE process

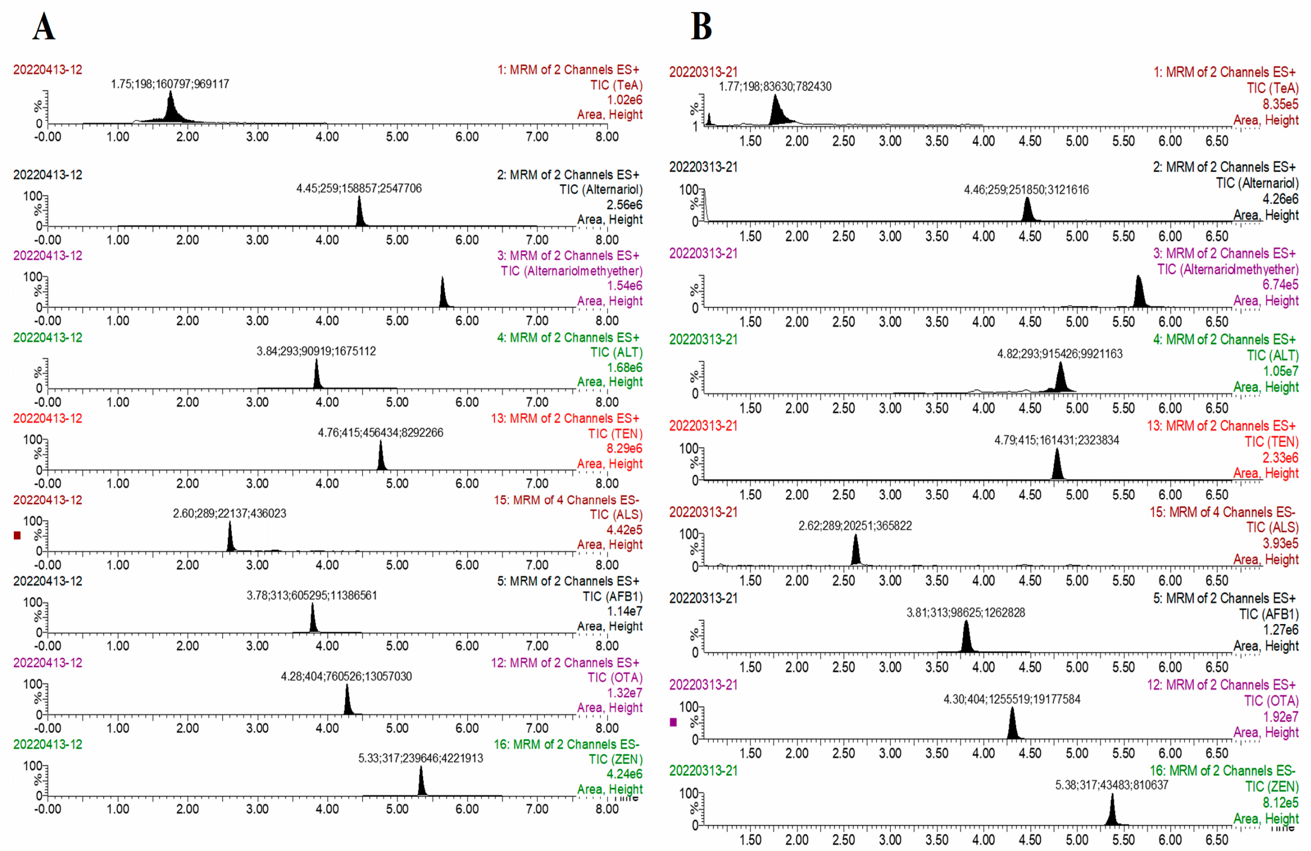

Figure S4. MRM chromatography of 9 mycotoxins in (A)solvent standard solution and (B) matrix standard solution (100 μg kg^{-1}). The retention time of peak corresponding to each toxin were as follow: TeA (1.75), AOH (4.45), AME (5.65), ALT (3.64), TEN (4.76), ALS (2.62), AFB$_1$ (3.78), OTA (4.28), ZEN (5.33)

References

1. Zhao, Y.; Wan, L.H.; Bai, X.L.; Liu, Y.M.; Zhang, F.P.; Liu, Y.M.; Liao, X. Quantification of mycotoxins in vegetable oil by UPLC-MS/MS after magnetic solid-phase extraction. *Food Addit. Contam.* **2017**, *34*, 1201–1210. https://doi.org/10.1080/19440049.2017.1319074.

2. Xu, H.W.; Sun, J.D.; Wang, H.M.; Zhang, Y.Z.; Sun, X.L. Adsorption of aflatoxins and ochratoxins in edible vegetable oils with dopamine-coated magnetic multi-walled carbon nanotubes. *Food Chem.* **2021**, *365*, 130409. https://doi.org/10.1016/j.foodchem.2021.130409.
3. Li, W.K.; Zhang, H.X.; Shi, Y.P. Simultaneous determination of aflatoxin B_1 and zearalenone by magnetic nanoparticle filled amino-modified multi-walled carbon nanotubes. *Anal. Methods* **2018**, *10*, 3353–3363. https://doi.org/10.1039/C8AY00815A.
4. Tang, Z.T.; Han, Q.R.; Yu, G.; Liu, F.; Tan, Y.Z.; Peng, C. Fe_3O_4@PDA/MIL-101(Cr) as magnetic solid-phase extraction sorbent for mycotoxins in licorice prior to ultrahigh-performance liquid chromatography-tandem mass spectrometry analysis. *Food Sci. Nutr.* **2022**, *10*, 2224–2235. https://doi.org/10.1002/fsn3.2832.
5. Wang, C.; Fan, Y.; He, W.; Hu, D.; Wu, A.; Wu, W. Development and Application of a QuEChERS-Based Liquid Chromatography Tandem Mass Spectrometry Method to Quantitate Multi-Component Alternaria Toxins in Jujube. *Toxins* **2018**, *10*, 382. https://doi.org/10.3390/toxins10100382.
6. De Berardis, S.; De Paola, E.L.; Montevecchi, G.; Garbini, D.; Masino, F.; Antonelli, A.; Melucci, D. Determination of four alternaria alternata mycotoxins by QuEChERS approach coupled with liquid chromatography-tandem mass spectrometry in tomato-based and fruit-based products. *Food Res. Int.* **2018**, *106*, 677–685. https://doi.org/10.1016/j.foodres.2018.01.032.
7. Xing, L.J.; Zou, L.J.; Luo, R.F.; Wang, Y. Determination of five Alternaria toxins in wolfberry using modified QuEChERS and ultra-high performance liquid chromatography-tandem mass spectrometry. *Food Chem.* **2020**, *311*, 125975. https://doi.org/10.1016/j.foodchem.2019.125975.
